# Supplementary material for: High levels of histones promote whole-genome-duplications and trigger a Swe1WEE1-dependent phosphorylation of Cdc28CDK1
Source: eLife. 2018 Mar 27;7:e35337. doi: 10.7554/eLife.35337 (PMC5871333; doi:10.7554/eLife.35337)
Supplement: Figure 1—source data 1. [file elife-35337-fig1-data1.pdf]

RAW DATA FOR THE ESTIMATION OF WGDs

experiment 1

		nº of colonies observed				
		day 3	day 5	WGDs at day 3 (n=10)	day3/day5 (%)	WGD %
wild type	vector	267	534	0/10	50,0	0,0
	CENΔNEG	147	252	0/10	58,3	0,0
rad53k227A	vector	715	856	0/10	83,5	0,0
	CENΔNEG	29	991	9/10	2,9	2,6

experiment 2

		nº of colonies observed				
		day 3	day 5	WGDs at day 3 (n=10)	day3/day5 (%)	WGD %
wild type	vector	350	560	0/10	62,5	0,0
	CENΔNEG	460	712	0/10	64,6	0,0
rad53k227A	vector	243	657	0/10	37,0	0,0
	CENΔNEG	27	1145	10/10	2,4	2,4
tom1Δ	vector	780	945	0/10	82,5	0,0
	CENΔNEG	46	1134	10/10	4,1	4,1

experiment 1

		day 3	day 5	WGDs at day 3 (n=10)	day3/day5 (%)	WGD %
wild type	vector	340	783	0/10	43,4	0,0
	2µΔNEG	12	651	7/10	1,8	1,3

experiment 2

		day 3	day 5	WGDs at day 3 (n=10)	day3/day5 (%)	WGD %
wild type	vector	340	632	0/10	53,8	0,0
	2µΔNEG	8	590	7/8	1,4	1,2

Source data Figure 1b. Raw data used to estimate the frequency of WGDs in rad53K227A and tom1Δ mutants after transformation with the centromeric version of the HTA1-HTB1ΔNEG vector or the frequency of wild type cells transformed with the 2µΔNEG vector. Methods used to estimate frequency can be found in the Material and Methods section.

		HTB1/TUB1 relative mRNA level				
		BIOLOGICAL REPLICATE	RAW DATA	AVERAGE	SD	paired t-test
wild type	vector	1	1,00	0,94	0,07	0,02927212
		2	0,94			
		3	0,87			
	2µΔNEG	1	1,41	1,63	0,24	
		2	1,60			
		3	1,87			

Source data Figure 1e. Q-PCR relaitve levels obtained for HTB1 mRNA in asynchronous haploid wild type cells transformed with an empty vector or the 2µΔNEG vector.

		duplication time (min)				
		replicate 1	replicate 2	Average	SD	SEM
wild type	vector	123	132	128	6	4
	CENΔNEG	158	170	164	8	6
rad53K227A	vector	130	133	131	2	2
	CENΔNEG (n)	225	202	213	16	11
	CENΔNEG (2n)	146	152	149	4	3

Source data Figure-Figure Supplement 1c: Duplication time (minutes) obtained in each of the two twelve hour kinetics performed for each strain and each vector.2n indicates strains that were completely diploid at the beggining and the end of the kinetic.

		duplication time (min)					
	EXPERIMENT	1	2	3	AVERAGE	SD	SEM
haploid (n)	vector	118	119	127	121	5	3
	2µΔNEG	275	246	228	250	24	14
diploid (2n)	vector	105	107	113	108	4	2
	2µΔNEG	180	195	160	178	18	10

Source data Figure-Figure Supplement 1f: Duplication time (minutes) obtained in each of the three twelve hour kinetics performed for each strain and each vector.
