# Supplementary material for: High levels of histones promote whole-genome-duplications and trigger a Swe1WEE1-dependent phosphorylation of Cdc28CDK1
Source: eLife. 2018 Mar 27;7:e35337. doi: 10.7554/eLife.35337 (PMC5871333; doi:10.7554/eLife.35337)
Supplement: Figure 2—source data 1. [file elife-35337-fig2-data1.pdf]

	RAW DATA						PERCENTAGE					
	VECTOR			*2µΔNEG*			VECTOR			*2µΔNEG*		
	A	B	C	1	2	3	A	B	C	1	2	3
**G1**	111	191	135	426	265	351	39,9	45,8	46,6	66,1	65,8	62,0
**bud and 1 SPB**	67	61	55	32	34	64	24,1	14,6	19,0	5,0	8,4	11,3
**bud 2 SPBs and 1 kinetochore**	35	65	40	95	64	99	12,6	15,6	13,8	14,8	15,9	17,5
**bud 2 SPBs and 2 kinetochores**	46	61	50	69	22	43	16,5	14,6	17,2	10,7	5,5	7,6
**bud 2 SPBs and 2 kinetochores no tubulin filament**	19	39	10	22	18	9	6,8	9,4	3,4	3,4	4,5	1,6
**n (each biological sample)**	278	417	290	644	403	566						
**n (each condition)**	985			1613								

	AVERAGE		SD		
	VECTOR	*2µΔNEG*	VECTOR	*2µΔNEG*	T-TEST PAIRED SAMPLES
**No bud**	44,1	64,6	3,63	2,28	0,022
**bud and 1 SPB**	19,2	8,2	4,74	3,17	0,115
**bud 2 SPBs and 1 kinetochore**	14,0	16,0	1,51	1,38	0,173
**bud 2 SPBs and 2 kinetochores**	16,1	7,9	1,35	2,64	0,021
**bud 2 SPBs and 2 kinetochores no tubulin filament**	6,5	3,2	2,96	1,46	0,061

Source data Figure 2b. Raw data obtained in each of the three experiment performed to obtain the numbers represented in figure 2b

	vector		*2µΔNEG*	
**DESCRIPTION**	**raw data**	**%**	**raw data**	**%**
**Normal mitosis (nucleus in the mother at the onset of anaphase; nuclei in mother and daughter at the end)**	181	97	168	73
**Mitosis starts in daughter cells (like type 1 but nucleus at the bud at the onset of anaphase)**	0	0	18	8
**Binucleation in bud: nuclei divide and remain in the bud. Cytokinesis takes place**	0	0	2	1
**Cells seem to try to divide their nucleus near the bud neck but don´t divide in at least 2 hours**	6	3	41	18
n	187		229	

Source data Figure 2c. Data corresponding to the quantification of the different types of cell divisions observed in the films performed with the first biological replicate.
