# Supplementary material for: High levels of histones promote whole-genome-duplications and trigger a Swe1WEE1-dependent phosphorylation of Cdc28CDK1
Source: eLife. 2018 Mar 27;7:e35337. doi: 10.7554/eLife.35337 (PMC5871333; doi:10.7554/eLife.35337)
Supplement: Figure 3—source data 1. [file elife-35337-fig3-data1.pdf]

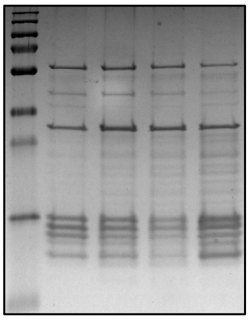

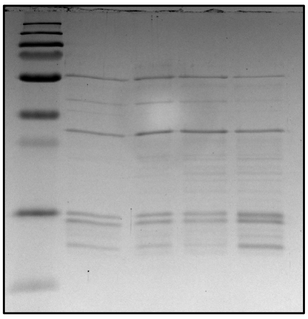

1st experiment		NON-SPECIFIC (NS) BAND	HISTONE SIGNAL (HS)	HS/NS	relative to rad53-AID control
rad53-AID	-	8.466.648	26.932.877	3,18	1,00
	NAA	10.735.648	29.300.635	2,73	0,86
rad53-AID lsm1Δ	-	9.438.234	21.198.271	2,25	0,71
	NAA	9.051.527	34.409.999	3,80	1,20

2nd experiment		NON-SPECIFIC (NS) BAND	HISTONE SIGNAL (HS)	HS/NS	relative to rad53-AID control
rad53-AID	-	9.246.062	16.259.371	1,76	1,00
	NAA	9.897.355	17.414.421	1,76	1,00
rad53-AID lsm1Δ	-	10.742.355	14.898.250	1,39	0,79
	NAA	9.068.770	22.048.735	2,43	1,38

t-test (paired, 2 tails)

		rad53-AID		***rad53-AID lsm1Δ***	
		**-**	**NAA**	**-**	**NAA**
rad53-AID	**-**	-	0.501	0.259	0.026
	**NAA**	-	-	0.082	0.144
rad53-AID lsm1Δ	**-**	-	-	-	0.124
	**NAA**	-	-	-	-

Source data Figure 3a. CBR staining for each of the two experiments in which core histones have been purified from rad53-AID and rad53-AID lsm1Δ cells treated or not with NAA and synchronized with Nocodazole. Quantification and statistics are shown below.

	**% of diploid cells**			
	**replicate 1**		**replicate 2**	
	**DMSO**	**NAA**	**DMSO**	**NAA**
haploid	100	90	100	70
diploid	0	10	0	5
rad53-AID lsm1Δ	0	10	0	6,66
**n**	100	100	75	75

**t-test (2 tails, unequal variance)**	0,038

Source data Figure 3g. Number of diploids observed by FACS in rad53-AID lsm1Δ cells after a 4 hour treatment with DMSO or NAA. This experiment was performed twice. In the first experiment, we counted 100 colonies for each condition. In the second one, we counted 75.

1st experiment	number of colonies		
	expected	counted	survival (NAA/untreated) %
rad53-AID untreated	100	108	80,6
	200	191	91,1
rad53-AID+NAA	100	87	
	200	174	
rad53-AID lsm1Δ untreated	100	68	2,9
	200	101	3,0
rad53-AID lsm1Δ+NAA	100	2	
	200	3	

t-test (paired, two tails)	0,0006

	number of colonies		
2nd experiment	expected	counted	survival (NAA/untreated) %
rad53-AID untreated	100	77	124,7
	200	124	106,5
rad53-AID+NAA	100	96	
	200	132	
rad53-AID lsm1Δ untreated	100	40	17,5
	200	72	12,5
rad53-AID lsm1Δ+NAA	100	7	
	200	9	

Source data Figure 3-Figure Supplement 3d. Relative colony formation assay data used to determine the number of viable cells after a four-hour treatment with NAA in *rad53-AID* and *rad53-AID lsm1*Δ mutants. % of viable cells is estimated dividing the number of viable cells obtained between in treated extracts divided by the number obtained in non-treated samples.

		biological replicate	RED(viable cells)	GREEN (dead cells) (%)	n
wild type	vector	1	99,0	1,0	306
		2	99,2	0,8	257
		3	95,7	3,0	300
	2µΔNEG	1	92,9	7,1	240
		2	89,2	10,8	204
		3	94,7	5,3	227

		Nº of dead cells (%)		
		AVERAGE	SD	SEM
wild type	vector	1,6	1,2	0,7
	2µΔNEG	7,7	2,8	1,6
t-test (paired, two tails)		0,11		

Source data Figure 3-Figure Supplement 3e. % of green cells (dead cells, unable to metabolise the compound) compared to red cells (able to metabolise the compound) observed in three independent experiments in wild type cells transformed with an empty vector or the 2µΔNEG vector.

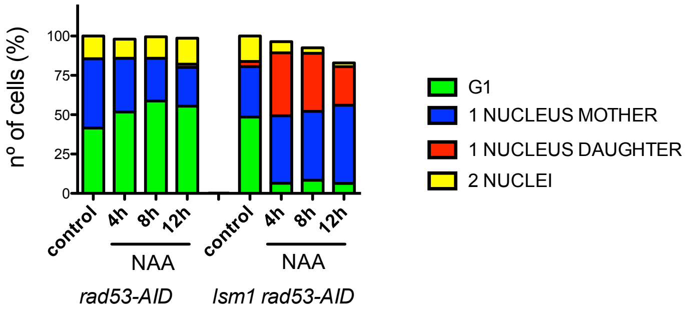

	1st experiment (%)				2nd experiment (%)			
rad53-AID	AS	4H NAA	8H NAA	12H NAA	AS	4H NAA	8H NAA	12H NAA
G1	35,4	38,8	45,8	80,6	41,5	51,8	58,8	55,4
bud, 1 nucleus in mother	45,5	48,8	38,3	12,2	44,0	34,1	27,0	24,6
bud, 1 nucleus in daughter	0,0	0,0	0,0	0,0	0,0	0,0	0,0	2,2
bud, two nuclei	19,2	11,9	15,4	5,9	14,5	12,2	13,7	16,5
n	198	201	201	222	207	164	204	224

	1st experiment (%)				2nd experiment (%)			
rad53-AID lsm1Δ	AS	4H NAA	8H NAA	12H NAA	AS	4H NAA	8H NAA	12H NAA
G1	23,4	5,5	7,0	9,2	48,6	6,4	8,4	6,3
bud, 1 nucleus in mother	44,0	47,6	42,2	44,2	31,9	42,9	43,8	49,7
bud, 1 nucleus in daughter	19,6	46,3	40,6	35,0	3,3	40,0	36,9	24,6
bud, two nuclei	10,5	0,0	0,8	2,5	16,2	7,1	3,4	2,3
n	209	164	128	120	210	140	203	175

Source data Figure 3-Figure Supplement 3f. Raw data obtained in the two cell cycle kinetics performed to determine changes in the cell cycle distribution of *rad53-AID* and *rad53-AID lsm1*Δ cells after treatment with NAA. Cells were fixed, stained with DAPI and classified according to morphology and number of nuclei using a 100x objective. The image on the top represents the numbers obtained in the second experiment, not included in the main text.
