# Supplementary material for: High levels of histones promote whole-genome-duplications and trigger a Swe1WEE1-dependent phosphorylation of Cdc28CDK1
Source: eLife. 2018 Mar 27;7:e35337. doi: 10.7554/eLife.35337 (PMC5871333; doi:10.7554/eLife.35337)
Supplement: Figure 4—source data 1. [file elife-35337-fig4-data1.pdf]

% IP HISTONE H3	*rad53-AID CONTROL (1)*			*rad53-AID TREATED (2)*			*lsm1 rad53-AID CONTROL (3)*			*lsm1 rad53-AID TREATED (4)*		
BIOLOGICAL REPLICATE	*1*	*2*	*3*	*1*	*2*	*3*	*1*	*2*	*3*	*1*	*2*	*3*
CEN4	9,24E-03	7,22E-03	1,89E-02	8,20E-03	2,13E-02	2,63E-02	3,66E-02	5,42E-02	5,93E-02	1,50E-01	2,47E-01	3,05E-01
CEN12	1,43E-02	6,99E-03	1,78E-02	6,74E-03	1,43E-02	2,00E-02	3,48E-02	2,30E-02	6,52E-02	1,64E-01	2,37E-01	3,55E-01
INT IV	4,26E-02	4,32E-02	8,59E-02	2,88E-02	4,76E-02	8,91E-02	5,55E-02	3,96E-02	1,01E-01	3,20E-01	3,64E-01	7,14E-01
INT XII	1,43E-01	1,17E-01	2,62E-01	1,06E-01	1,84E-01	2,99E-01	1,21E-01	9,46E-02	2,38E-01	2,83E-01	4,09E-01	7,07E-01
ALG9	1,29E-01	1,13E-01	2,62E-01	9,07E-02	1,54E-01	2,50E-01	1,29E-01	1,02E-01	2,24E-01	2,76E-01	4,37E-01	6,25E-01

% IP HISTONE H4	*rad53-AID CONTROL (1)*			*rad53-AID TREATED (2)*			*lsm1 rad53-AID CONTROL (3)*			*lsm1 rad53-AID TREATED (4)*		
BIOLOGICAL REPLICATE	*1*	*2*	*3*	*1*	*2*	*3*	*1*	*2*	*3*	*1*	*2*	*3*
CEN4	2,04E-02	3,02E-02	2,78E-02	1,92E-02	4,20E-02	2,90E-02	5,94E-02	5,27E-02	4,85E-02	9,85E-02	1,56E-01	1,28E-01
CEN12	2,97E-02	2,86E-02	2,95E-02	1,81E-02	3,66E-02	2,49E-02	5,10E-02	4,07E-02	3,94E-02	1,01E-01	1,84E-01	1,35E-01
INT IV	9,99E-02	1,29E-01	1,30E-01	8,10E-02	1,27E-01	9,38E-02	1,37E-01	1,02E-01	1,06E-01	1,53E-01	2,26E-01	2,13E-01
INT XII	2,26E-01	2,49E-01	3,30E-01	2,07E-01	2,84E-01	2,29E-01	2,65E-01	2,14E-01	2,08E-01	1,80E-01	2,66E-01	2,35E-01
ALG9	1,95E-01	2,20E-01	3,18E-01	2,19E-01	2,50E-01	2,12E-01	2,71E-01	1,93E-01	2,18E-01	1,71E-01	2,68E-01	2,30E-01

% IP CENP-A	*rad53-AID CONTROL (1)*			*rad53-AID TREATED (2)*			*lsm1 rad53-AID CONTROL (3)*			*lsm1 rad53-AID TREATED (4)*		
BIOLOGICAL REPLICATE	*1*	*2*	*3*	*1*	*2*	*3*	*1*	*2*	*3*	*1*	*2*	*3*
CEN4	4,83E-02	5,94E-02	5,09E-02	5,97E-02	7,08E-02	6,63E-02	7,33E-02	6,17E-02	6,05E-02	4,91E-02	1,01E-01	7,69E-02
CEN12	4,41E-02	3,13E-02	2,20E-02	3,09E-02	3,22E-02	2,45E-02	4,68E-02	3,11E-02	3,14E-02	4,27E-02	9,95E-02	8,83E-02
INT IV	5,34E-04	9,57E-04	8,14E-04	4,40E-04	1,61E-03	1,10E-03	1,10E-03	1,02E-03	8,64E-04	1,27E-03	3,24E-03	2,45E-03
INT XII	6,93E-04	1,36E-03	1,83E-03	8,39E-04	3,18E-03	2,36E-03	1,21E-03	1,28E-03	1,64E-03	1,39E-03	3,40E-03	2,96E-03
ALG9	6,93E-04	1,36E-03	2,78E-03	7,40E-04	2,84E-03	2,56E-03	1,18E-03	1,52E-03	1,54E-03	1,47E-03	3,30E-03	3,68E-03

T-TEST PAIRED SAMPLES (2 TAILS)

HISTONE H3	1 VS 2	1 VS 3	**1 VS 4**	2 VS 4	3 VS 4
CEN4	0,260	0,022	**0,035**	0,032	0,041
CEN12	0,899	0,104	**0,048**	0,044	0,045
INT IV	0,755	0,304	**0,066**	0,062	0,065
INT XII	0,547	0,001	**0,080**	0,062	0,071
ALG9	0,914	0,295	**0,053**	0,036	0,061
HISTONE H4	1 VS 2	1 VS 3	**1 VS 4**	2 VS 4	3 VS 4
CEN4	0,428	0,042	**0,018**	0,011	0,059
CEN12	0,678	0,053	**0,045**	0,026	0,070
INT IV	0,203	0,841	**0,027**	0,019	0,134
INT XII	0,551	0,491	**0,334**	0,319	0,964
ALG9	0,737	0,769	**0,643**	0,869	0,943
CENP-A	1 VS 2	1 VS 3	**1 VS 4**	2 VS 4	3 VS 4
CEN4	0,01	0,21	**0,19**	0,48	0,63
CEN12	0,58	0,30	**0,19**	0,12	0,21
INT IV	0,32	0,32	**0,07**	0,03	0,16
INT XII	0,24	0,76	**0,08**	0,06	0,17
ALG9	0,49	0,75	**0,08**	0,06	0,13

% IP NO AB

	1	2	3
CEN4	5,30E-04	7,40E-04	5,50E-04
CEN12	5,57E-04	4,26E-04	3,71E-04
INT IV	2,93E-04	3,61E-04	2,67E-04
INT XII	3,91E-04	8,07E-04	5,55E-04
ALG9	4,62E-04	6,41E-04	3,63E-04

Source data Figure 4a. ChIP raw data represented in figure 4a. p-values obtained are also presented below

ASYNCHRONOUS CULTURES

% IP MTW1-mCH	*rad53-AID CONTROL (1)*			*rad53-AID TREATED (2)*			*lsm1 rad53-AID CONTROL (3)*			*lsm1 rad53-AID TREATED (4)*		
REPLICATE	1	2	3	1	2	3	1	2	3	1	2	3
CEN4	6,3623	6,1334	8,4952	6,4225	5,5030	9,2923	4,5754	5,2982	8,8098	7,9827	6,9326	6,7861
CEN12	1,6055	1,5633	4,3481	0,9640	1,3537	2,8005	1,5970	2,3445	3,4296	5,3580	5,6918	4,7060
INT IV	0,0000	0,0000	0,0048	0,0000	0,0000	0,0000	0,0000	0,0000	0,0210	0,0045	0,0000	0,0036
ALG9	0,0000	0,0032	0,0004	0,0036	0,0000	0,0000	0,0000	0,0000	0,0205	0,0000	0,0057	0,0000

T-TEST PAIRED SAMPLES (2 TAILS)

HISTONE H3	1 VS 2	1 VS 3	**1 VS 4**	2 VS 4	3 VS 4
CEN4	0,871	0,333	**0,835**	0,915	0,594
CEN12	0,180	0,930	**0,149**	0,050	0,068
INT IV	0,423	0,423	**0,584**	0,188	0,586
ALG9	0,995	0,522	**0,512**	0,817	0,598

SYNCHRONIZED CULTURES

% IP MTW1-mCH	*rad53-AID CONTROL (1)*		*rad53-AID TREATED (2)*		*lsm1 rad53-AID CONTROL (3)*		*lsm1 rad53-AID TREATED (4)*	
REPLICATE	1	2	1	2	1	2	1	2
CEN4	3,2020	3,6200	2,6611	1,8939	2,3730	1,4268	1,7445	2,7091
CEN12	1,0543	1,1403	0,7559	0,6520	0,6889	0,7057	1,5308	1,8551
INT IV	0,0010	0,0020	0,0006	0,0011	0,0010	0,0021	0,0017	0,0035
ALG9	0,0020	0,0020	0,0017	0,0015	0,0012	0,0031	0,0023	0,0022

T-TEST PAIRED SAMPLES (2 TAILS)

HISTONE H3	1 VS 2	1 VS 3	**1 VS 4**	2 VS 4	3 VS 4
CEN4	0,307	0,270	**0,144**	0,963	0,790
CEN12	0,151	0,055	**0,126**	0,136	0,098
INT IV	0,246	0,779	**0,230**	0,236	0,199
ALG9	0,173	0,897	**0,064**	0,080	0,935

Source data Figure 4b and c. ChIP raw data represented in figures 4b (up) and 4c (low). p-values obtained are also presented below.
