# Supplementary material for: High levels of histones promote whole-genome-duplications and trigger a Swe1WEE1-dependent phosphorylation of Cdc28CDK1
Source: eLife. 2018 Mar 27;7:e35337. doi: 10.7554/eLife.35337 (PMC5871333; doi:10.7554/eLife.35337)
Supplement: Figure 5—source data 1. [file elife-35337-fig5-data1.pdf]

% IP HISTONE H2B	*rad53-AID* CONTROL (1)			*rad53-AID* TREATED (2)			*lsm1 rad53-AID* CONTROL (3)			*lsm1 rad53-AID* TREATED (4)		
BIOLOGICAL REPLICATE	1	2	3	1	2	3	1	2	3	1	2	3
CEN4 L	1,04E-01	2,09E-01	2,95E-01	1,32E-01	1,78E-01	1,98E-01	1,79E-01	2,10E-01	2,59E-01	4,49E-01	5,08E-01	4,40E-01
CEN4 R	1,43E-01	2,13E-01	2,45E-01	2,03E-01	2,45E-01	1,81E-01	2,31E-01	2,67E-01	2,20E-01	4,29E-01	5,63E-01	4,62E-01
CEN12 L	9,98E-02	1,31E-01	2,18E-01	1,49E-01	1,55E-01	1,82E-01	2,21E-01	1,85E-01	2,69E-01	3,82E-01	5,76E-01	5,22E-01
CEN12 R	1,16E-01	1,60E-01	2,02E-01	1,28E-01	1,65E-01	1,59E-01	2,27E-01	2,47E-01	2,65E-01	4,15E-01	5,41E-01	5,83E-01
INT IV	1,29E-01	2,04E-01	2,76E-01	1,39E-01	1,57E-01	1,54E-01	2,24E-01	2,19E-01	2,93E-01	4,05E-01	5,89E-01	5,60E-01
INT XII	4,10E-01	5,34E-01	8,94E-01	5,23E-01	3,99E-01	5,56E-01	5,16E-01	2,52E-01	6,47E-01	4,57E-01	3,28E-01	5,70E-01
ALG9	3,44E-01	4,00E-01	6,08E-01	4,28E-01	4,26E-01	5,02E-01	4,15E-01	2,42E-01	5,85E-01	3,86E-01	5,70E-01	5,05E-01

T-TEST PAIRED SAMPLES

	1 VS 2	1 VS 3	**1 VS 4**	2 VS 4	3 VS 4
CEN4 L	0,455	0,719	**0,049**	0,009	0,020
CEN4 R	0,826	0,369	**0,018**	0,009	0,013
CEN12 L	0,676	0,083	**0,021**	0,026	0,057
CEN12 R	0,648	0,024	**0,006**	0,012	0,022
INT IV	0,298	0,248	**0,012**	0,019	0,038
INT XII	0,454	0,372	**0,278**	0,277	0,719
ALG9	0,984	0,635	**0,691**	0,595	0,627

% IP HISTONE H2A	*rad53-AID* CONTROL (1)			*rad53-AID* TREATED (2)			*lsm1 rad53-AID* CONTROL (3)			*lsm1 rad53-AID* TREATED (4)		
BIOLOGICAL REPLICATE	1	2	3	1	2	3	1	2	3	1	2	3
CEN4 L	1,86E-01	3,65E-02	7,15E-02	1,41E-01	5,27E-02	8,56E-02	2,17E-01	5,88E-02	7,24E-02	3,94E-01	2,20E-01	3,11E-01
CEN4 R	1,84E-01	3,83E-02	7,20E-02	1,05E-01	4,87E-02	6,74E-02	2,51E-01	7,18E-02	8,74E-02	4,27E-01	1,79E-01	3,24E-01
CEN12 L	1,34E-01	2,32E-02	4,07E-02	9,51E-02	5,22E-02	5,88E-02	1,90E-01	4,28E-02	5,15E-02	4,29E-01	2,26E-01	3,02E-01
CEN12 R	1,18E-01	2,83E-02	4,49E-02	8,18E-02	3,22E-02	5,42E-02	1,75E-01	4,72E-02	5,33E-02	3,92E-01	2,27E-01	2,89E-01
INT IV	1,89E-01	4,01E-02	6,05E-02	1,01E-01	4,64E-02	5,26E-02	2,30E-01	5,95E-02	7,23E-02	5,13E-01	2,17E-01	3,26E-01
INT XII	3,32E-01	8,64E-02	1,44E-01	2,94E-01	1,06E-01	1,24E-01	3,60E-01	1,58E-01	1,57E-01	5,44E-01	2,34E-01	3,32E-01

T-TEST PAIRED SAMPLES

	1 VS 2	1 VS 3	**1 VS 4**	2 VS 4	3 VS 4
CEN4 L	0,831	0,180	**0,006**	0,013	0,015
CEN4 R	0,471	0,123	**0,027**	0,053	0,043
CEN12 L	0,906	0,173	**0,011**	0,033	0,009
CEN12 R	0,647	0,196	**0,008**	0,018	0,006
INT IV	0,416	0,108	**0,027**	0,055	0,026
INT XII	0,534	0,164	**0,010**	0,032	0,053

% IP HISTONE H2A.Z	*rad53-AID* CONTROL (1)			*rad53-AID* TREATED (2)			*lsm1 rad53-AID* CONTROL (3)			*lsm1 rad53-AID* TREATED (4)		
BIOLOGICAL REPLICATE	1	2	3	1	2	3	1	2	3	1	2	3
CEN4 L	3,27E-02	2,63E-02	1,74E-02	2,28E-02	2,23E-02	9,58E-03	1,43E-02	2,05E-02	2,29E-02	6,16E-03	7,43E-03	5,98E-03
CEN4 R	2,43E-02	1,80E-02	2,57E-02	1,79E-02	1,71E-02	1,73E-02	1,64E-02	2,41E-02	7,59E-03	6,53E-03	8,25E-03	6,20E-03
CEN12 L	3,91E-02	3,17E-02	4,11E-02	3,43E-02	3,43E-02	3,07E-02	1,87E-02	2,04E-02	2,45E-02	6,47E-03	1,09E-02	5,56E-03
CEN12 R	3,06E-02	2,24E-02	2,88E-02	2,23E-02	2,35E-02	1,75E-02	1,73E-02	1,91E-02	1,64E-02	5,71E-03	6,70E-03	5,75E-03
INT IV	5,72E-02	5,47E-02	4,93E-02	4,63E-02	5,04E-02	4,06E-02	3,26E-02	4,32E-02	3,70E-02	1,03E-02	1,48E-02	9,09E-03
INT XII	1,94E-02	7,40E-03	1,62E-02	1,85E-02	1,99E-02	1,11E-02	1,40E-02	1,91E-02	1,23E-02	5,71E-03	8,91E-03	5,20E-03
ALG9	1,19E-02	1,24E-02	1,04E-02	9,49E-03	1,29E-02	6,55E-03	8,12E-03	6,20E-03	7,84E-03	6,26E-03	7,99E-03	5,30E-03

T-TEST PAIRED SAMPLES

	1 VS 2	1 VS 3	**1 VS 4**	2 VS 4	3 VS 4
CEN4 L	0,051	0,460	**0,049**	0,103	0,038
CEN4 R	0,143	0,445	**0,034**	0,006	0,164
CEN12 L	0,382	0,026	**0,022**	0,002	0,040
CEN12 R	0,242	0,095	**0,017**	0,012	0,002
INT IV	0,055	0,062	**0,003**	0,002	0,006
INT XII	0,725	0,899	**0,241**	0,041	0,011
ALG9	0,273	0,060	**0,005**	0,096	0,584

		NO AB	
CEN4 L	3,36E-04	4,96E-04	2,57E-04
CEN4 R	5,21E-04	4,17E-04	4,67E-04
CEN12 L	7,49E-04	5,95E-04	5,17E-04
CEN12 R	3,67E-04	8,80E-04	5,54E-04
INT IV	3,80E-04	5,57E-04	4,68E-04
INT XII	4,24E-04	8,36E-04	3,14E-04
ALG9	8,46E-04	7,88E-04	5,37E-04

Source data Figure 5a . ChIP raw data represented in figures 5a. p-values obtained are also presented below.

			SIGNAL QUANTIFICATION			
		BIOLOGICAL REPLICATE	H2B	ACT1 (H2B)	H2AZ	ACT1 (H2AZ)
wild type	vector	1	13365	20620	23190	18655
		2	7970	3767	22700	5608
		3	18621	23688	24155	23638
	2μΔNEG	1	20524	32855	20788	33570
		2	21869	30057	16679	29344
		3	17881	27717	26120	24385
		4	8407	20558	25660	21505
		5	23246	31367	29210	31716

		LEVELS RELATIVE TO ACT1 LOADING CONTROL		
		BIOLOGICAL REPLICATE	H2B	H2AZ
wild type	vector	1	0,65	0,93
	*	2	2,12	2,56
		3	0,79	0,76
	2μΔNEG	1	0,62	0,43
		2	0,73	0,40
		3	0,65	0,64
	**	4	0,41	0,53
		5	0,74	0,42

	AVERAGE	
	H2B	H2AZ
VECTOR	0,72	0,84
2-μΔNEG	0,68	0,47
	SD	
	H2B	H2AZ
VECTOR	0,10	0,12
2-μΔNEG	0,06	0,11

* EXCLUDED FROM QUANTIFICATION, VERY LOW LEVELS OF ACT1

** EXCLUDED FROM QUANTIFICATION, PARTIALLY DIPLOID IN FACS

Source data Figure 5c. Quantification of the signal obtained for histones H2B and H2A.Z in asynchronous wild type cells transformed with an empty vector or the 2μΔNEG vector.

number of cells (%)

	1ST EXP		2ND EXP		3RD EXP		4TH EXP		AVERAGE		SD	
	n	2n	n	2n	n	2n	n	2n	n	2n	n	2n
2μNEG+vector	98,52	**1,48**	97,85	**2,15**	98,90	**1,10**	99,44	**0,56**	98,42	**1,58**	0,53	0,53
2μNEG+HTZ1	99,21	**0,79**	99,22	**0,78**	99,11	**0,89**	99,76	**0,24**	99,18	**0,82**	0,06	0,06

t-test (paired 1 tail)	0,045

Source data Figure 5e. Quantification of the number of diploids observed in cells transformed simultaneously with the 2μΔNEG vector and an empty vector or with the first and plasmid pRS425-HTZ1.

		DAY 1			DAY 2			DAY 3			DAY 4		
		HAPLOID	MIXTURE	DIPLOID	HAPLOID	MIXTURE	DIPLOID	HAPLOID	MIXTURE	DIPLOID	HAPLOID	MIXTURE	DIPLOID
1ST EXP (n = 8)	*wild type*	8	0	0	6	2	0	2	6	0	2	2	4
	swr1Δ	0	0	8	0	0	8	0	0	8	0	0	8
2ND EXP (n = 7)	*wild type*	6	1	0	5	2	0	1	4	2	1	3	3
	swr1Δ	3	3	1	0	2	5	0	0	7	0	0	7
TOTAL (%)	*wild type*	14	1	0	11	4	0	3	10	2	3	5	7
	swr1Δ	3	3	9	0	2	13	0	0	15	0	0	15
	wild type	93	7	0	73	27	0	20	67	13	20	33	47
	swr1Δ	20	20	60	0	13	87	0	0	100	0	0	100

Source data Figure 5f: Quantification of the number of haploids, diploids and cells in which a mixed profile can be observed in wild type cells or *swr1*Δ cells transformed with the 2μΔNEG. Two independent experiments were performed. Experiment 1 was carried out with 8 independent colonies for each background and also carried two candidates transformed with an empty vector that were measured only on day 1 and day 4. Experiment 2 was performed with 7 independent colonies and two empty vectors for each genetic background. All cells trasnformed with the empty vector were haploid at day 1 and 4.

	rad53-AID CONTROL					rad53-AID TREATED				
	1	2	3	4	5	1	2	3	4	5
CEN4 L	5,74E-04	4,34E-04	6,86E-01	6,06E-01	1,46E+00	1,03E-03	2,23E-04	5,40E-01	3,24E-01	8,52E-01
CEN4 R	5,39E-04	4,38E-04	6,25E-01	1,53E-01	1,77E-01	1,18E-03	2,13E-04	3,17E-01	1,79E-01	2,59E-01
CEN12L	8,60E-04	6,90E-04	1,17E+00	3,45E-01	4,93E-01	1,10E-03	4,01E-04	8,83E-01	3,37E-01	6,45E-01
CEN12R	6,46E-04	5,21E-04	1,13E+00	2,68E-01	4,21E-01	1,10E-03	3,23E-04	7,31E-01	2,92E-01	4,63E-01

	lsm1 rad53-AID CONTROL					lsm1 rad53-AID TREATED					NO TAG	
	1	2	3	4	5	1	2	3	4	5	1	2
CEN4 L	2,27E-04	1,54E-04	9,27E-01	6,70E-01	4,89E-01	9,70E-05	3,02E-05	8,22E-01	6,53E-01	1,74E+00	2,38E-06	3,76E-06
CEN4 R	1,59E-04	1,28E-04	2,18E-01	3,36E-02	9,11E-02	8,29E-05	3,68E-05	2,72E-02	2,95E-02	1,13E-02	1,10E-06	1,25E-06
CEN12L	3,17E-04	1,24E-04	3,26E-01	6,01E-02	1,51E-01	1,24E-04	7,06E-05	3,21E-02	4,16E-02	1,10E-02	2,73E-06	2,82E-06
CEN12R	2,27E-04	1,29E-04	2,73E-01	5,20E-02	1,25E-01	1,17E-04	5,60E-05	2,94E-02	2,71E-02	1,05E-02	1,89E-06	0,00E+00

T-TEST PAIRED SAMPLES (1 tail)

	1 VS 2	1 VS 3	**1 VS 4**	2 VS 4	3 VS 4
CEN4 L	0,071	0,284	**0,079**	0,070	0,215
CEN4 R	0,296	0,088	**0,091**	0,042	0,107
CEN12 L	0,356	0,064	**0,070**	0,052	0,094
CEN12 R	0,235	0,078	**0,079**	0,051	0,088

Source data Figure 5g. ChIP raw data represented in figure 5g for Brn1-HA ChIP. P-values and all statistical tests performed are indicated.

	rad53-AID lsm1Δ prs425 CONTROL (1)			*rad53-AID lsm1Δ* prs425 TREATED (2)			*rad53-AID lsm1Δ* prs425-HTZ1 CONTROL (3)			*rad53-AID lsm1Δ* prs425-HTZ1 TREATED (4)		
	1	2	3	1	2	3	1	2	3	1	2	3
CEN4 L	1,67E-01	1,07E-01	6,10E-02	2,99E-02	4,15E-02	4,95E-02	3,49E-02	2,95E-02	8,72E-02	3,87E-02	7,62E-02	8,70E-02
CEN4 R	3,31E-02	2,81E-02	3,23E-02	1,28E-02	1,03E-02	1,50E-02	2,56E-02	1,41E-02	2,06E-02	7,39E-03	5,81E-03	9,25E-03
CEN12 L	3,73E-02	2,95E-02	3,07E-02	8,69E-03	9,24E-03	1,32E-02	2,02E-02	1,53E-02	2,27E-02	1,12E-02	6,30E-03	9,06E-03
CEN12 R	2,77E-02	1,77E-02	1,39E-02	7,21E-03	1,17E-02	1,64E-02	2,29E-02	1,30E-02	1,61E-02	6,71E-03	7,21E-03	9,81E-03
INT IV	6,20E-02	4,26E-02	3,93E-02	1,22E-02	1,25E-02	1,83E-02	2,65E-02	2,25E-02	1,74E-02	7,00E-03	7,71E-03	1,21E-02
INT XII	2,09E-02	2,03E-02	1,69E-02	5,98E-03	9,00E-03	1,55E-02	1,70E-02	2,10E-02	2,45E-02	9,33E-03	1,04E-02	8,69E-03

T-TEST PAIRED SAMPLES (1 tail)

	**1 VS 2**	1 VS 3	1 VS 4	2 VS 4	**3 VS 4**
CEN4 L	**0,094**	0,159	0,214	0,049	**0,191**
CEN4 R	**0,001**	0,014	0,001	0,003	**0,025**
CEN12 L	**0,011**	0,020	0,002	0,265	**0,010**
CEN12 R	**0,177**	0,198	0,069	0,082	**0,054**
INT IV	**0,029**	0,017	0,021	0,003	**0,043**
INT XII	**0,075**	0,353	0,005	0,422	**0,020**

Source data Figure 5-Figure Supplement 5c. ChIP raw data represented in figure 5c for H2A.Z ChIP. p-values and all statistical tests performed are indicated.
