# Supplementary material for: High levels of histones promote whole-genome-duplications and trigger a Swe1WEE1-dependent phosphorylation of Cdc28CDK1
Source: eLife. 2018 Mar 27;7:e35337. doi: 10.7554/eLife.35337 (PMC5871333; doi:10.7554/eLife.35337)
Supplement: Figure 6—source data 1. [file elife-35337-fig6-data1.pdf]

	1st experiment				2nd experiment			
	rad53-AID		rad53-AID lsm1		rad53-AID		rad53-AID lsm1	
RAW DATA	untreated	NAA	untreated	NAA	untreated	NAA	untreated	NAA
MAD2 FOCI-MTW1 FOCI	1	0	0	1	0	1	0	0
MAD2 FOCI ALONE	0	0	0	0	0	0	0	0
NO MAD2 FOCI	155	126	122	157	104	115	120	112
n	156	126	122	158	104	116	120	112
PERCENTAGE								
MAD2 FOCI-MTW1 FOCI	0,6	0,0	0,0	0,6	0,0	0,9	0,0	0,0
MAD2 FOCI ALONE	0,0	0,0	0,0	0,0	0,0	0,0	0,0	0,0
NO MAD2 FOCI	99,4	100,0	100,0	99,4	100,0	99,1	100,0	100,0

Source data Figure 6b. Raw data for the quantification of Mad2 foci.

		% IP					
		CEN4 R	CEN4 L	INT IV	CEN12L	CEN12 R	INT XII
1st experiment	MET	8,49E-03	1,01E-02	4,76E-04	2,27E-02	1,21E-02	1,19E-03
	MET+NOC	7,64E-02	5,63E-02	1,78E-03	3,19E-01	7,19E-02	7,72E-03
	MET+NAA	1,11E-02	1,33E-02	2,56E-04	2,18E-02	1,44E-02	1,06E-03
2nd experiment	MET	2,47E-02	1,64E-02	3,20E-03	3,97E-02	2,97E-02	4,62E-03
	MET+NOC	4,45E-02	5,21E-02	1,91E-03	8,93E-02	6,14E-02	3,99E-03
	MET+NAA	3,43E-02	2,08E-02	1,01E-02	4,72E-02	4,14E-02	1,47E-02

	STUDENT T-TEST PAIRED SAMPLES (2 TAILS)					
	CEN4 R	CEN4 L	INT IV	CEN12L	CEN12 R	INT XII
MET VS NOC	0,32	0,08	1,00	0,39	0,19	0,56
MET VS NAA	0,33	0,10	0,52	0,58	0,38	0,51
NOC VS NAA	0,40	0,10	0,62	0,41	0,29	0,85

	STUDENT T-TEST PAIRED (1 TAIL)					
	CEN4 R	CEN4 L	INT IV	CEN12L	CEN12 R	INT XII
MET VS NOC	0,16	0,04	0,50	0,20	0,09	0,28
MET VS NAA	0,17	0,05	0,26	0,29	0,19	0,25
NOC VS NAA	0,20	0,05	0,31	0,21	0,14	0,43

Source data Figure 6c. Raw data for the ChIP experiment to measure Sgo1-HA recruitment. Two independent biological replicates.p-values and statistical tests used to obtain them are indicated.
