# Supplementary material for: High levels of histones promote whole-genome-duplications and trigger a Swe1WEE1-dependent phosphorylation of Cdc28CDK1
Source: eLife. 2018 Mar 27;7:e35337. doi: 10.7554/eLife.35337 (PMC5871333; doi:10.7554/eLife.35337)
Supplement: Figure 7—source data 1. [file elife-35337-fig7-data1.pdf]

			DDC2 FOCI (%)	n		N	AVERAGE	SD	SEM
BIOLOGICAL REPLICATE 1	VECTOR	colony 1	5,6	213	VECTOR	1065	6,17	0,41	0,20
		colony 2	6,3	301	2µ-NEG	1068	6,15	1,97	0,99
	2µ-NEG	colony 1	6,1	314					
		colony 2	4,1	98	T-TEST PAIRED SAMPLES			0,9844	
BIOLOGICAL REPLICATE 2	VECTOR	colony 1	6,2	307					
		colony 2	6,6	244					
	2µ-NEG	colony 1	5,6	463					
		colony 2	8,8	193					

Source data Figure 7b. Raw data for the quantification of Ddc2 foci. Two independent experiments with two different colonies analysed on each experiment.

TYPES	
A	CLEAR SPOT OF MAD2 THAT COLOCALIZES WITH SIGNAL OF MTW1
B	PARTIAL CO-LOCALISATION, COLOCALISATION BUT VERY WEAK MAD2 SIGNAL
C	WEAK SPOTS OF MAD2 THAT DO NOT CO-LOCALIZE

			NUMBER OF EVENTS PER MIL ‰					type A events			
			A	B	C	n		N	AVERAGE	SD	SEM
BIOLOGICAL REPLICATE 1	VECTOR	colony 1	3,3	6,5	3,3	306	VECTOR	2260	4,6	3,7	1,85
		colony 2	7,6	0	0	264	2µ-NEG	2500	9,3	1,4	0,7
	2µ-NEG	colony 1	9,1	9,1	0	660					
		colony 2	9,7	9,7	1,9	515	t-test (paired samples, two tails)	0,0311072			
BIOLOGICAL REPLICATE 2	VECTOR	colony 1	7,6	15,3	7,6	262					
		colony 2	0	7,9	0	253					
	2µ-NEG	colony 1	10,9	0	0	275					
		colony 2	7,5	9,3	3,7	535					

Source data Figure 7c. Raw data for the quantification of Mad2 foci. Two independent experiments with two different colonies analysed on each experiment. The representation observed in figure 7c only includes Mad2 foci that clearly colocalised to Mtw1 foci (Type A events).
