# Supplementary material for: High levels of histones promote whole-genome-duplications and trigger a Swe1WEE1-dependent phosphorylation of Cdc28CDK1
Source: eLife. 2018 Mar 27;7:e35337. doi: 10.7554/eLife.35337 (PMC5871333; doi:10.7554/eLife.35337)
Supplement: Figure 8—source data 1. [file elife-35337-fig8-data1.pdf]

2µΔNEG

		DAY 1			DAY 2			DAY 3			DAY 4		
		**HAPLOID**	**MIXTURE**	**DIPLOID**	**HAPLOID**	**MIXTURE**	**DIPLOID**	**HAPLOID**	**MIXTURE**	**DIPLOID**	**HAPLOID**	**MIXTURE**	**DIPLOID**
	wild type	4	0	0	1	3	0	0	1	3	1	1	2
	swe1Δ	1	2	1	0	0	4	0	0	4	0	0	4
**FIRST REP (n = 4)**	***mad2Δ***	0	0	4	0	0	4	0	0	4	0	0	4
	wild type	4	0	0	1	3	0	1		3	1	1	2
	swe1Δ	1	1	2	0	0	4	0	0	4	0	0	4
**SECOND REP (n = 4)**	***mad2Δ***	0	0	4	0	0	4	0	0	4	0	0	4

empty vector

		DAY 1			DAY 2			DAY 3			DAY 4		
		**HAPLOID**	**MIXTURE**	**DIPLOID**	**HAPLOID**	**MIXTURE**	**DIPLOID**	**HAPLOID**	**MIXTURE**	**DIPLOID**	**HAPLOID**	**MIXTURE**	**DIPLOID**
	wild type	2	0	0	-	-	-	-	-	-	2	0	0
	swe1Δ	2	0	0	-	-	-	-	-	-	2	0	0
**FIRST REP (n = 4)**	***mad2Δ***	2	0	0	-	-	-	-	-	-	2	0	0
	wild type	2	0	0	-	-	-	-	-	-	2	0	0
	swe1Δ	2	0	0	-	-	-	-	-	-	2	0	0
**SECOND REP (n = 4)**	***mad2Δ***	1	0	0	-	-	-	-	-	-	1	0	0

2µΔNEG (IN %)

PERCENTAGE		DAY 1			DAY 2			DAY 3			DAY 4		
		**HAPLOID**	**MIXTURE**	**DIPLOID**	**HAPLOID**	**MIXTURE**	**DIPLOID**	**HAPLOID**	**MIXTURE**	**DIPLOID**	**HAPLOID**	**MIXTURE**	**DIPLOID**
	wild type	100	0	0	25	75	0	0	25	75	25	25	50
	swe1Δ	25	50	25	0	0	100	0	0	100	0	0	100
**FIRST REP (n = 4)**	***mad2Δ***	0	0	100	0	0	100	0	0	100	0	0	100
	wild type	100	0	0	25	75	0	25	0	75	25	25	50
	swe1Δ	25	25	50	0	0	100	0	0	100	0	0	100
**SECOND REP (n = 4)**	***mad2Δ***	0	0	100	0	0	100	0	0	100	0	0	100

2µΔNEG (AVERAGE, IN %)

AVERAGE		DAY 1			DAY 2			DAY 3			DAY 4		
		**HAPLOID**	**MIXTURE**	**DIPLOID**	**HAPLOID**	**MIXTURE**	**DIPLOID**	**HAPLOID**	**MIXTURE**	**DIPLOID**	**HAPLOID**	**MIXTURE**	**DIPLOID**
	wild type	100	0	0	25	75	0	13	13	75	25	25	50
	swe1Δ	25	38	38	0	0	100	0	0	100	0	0	100
	mad2Δ	0	0	100	0	0	100	0	0	100	0	0	100

2µΔNEG (SD, IN %)

SD		DAY 1			DAY 2			DAY 3			DAY 4		
		**HAPLOID**	**MIXTURE**	**DIPLOID**	**HAPLOID**	**MIXTURE**	**DIPLOID**	**HAPLOID**	**MIXTURE**	**DIPLOID**	**HAPLOID**	**MIXTURE**	**DIPLOID**
	wild type	0	0	0	0	0	0	18	18	0	0	0	0
	swe1Δ	0	18	18	0	0	0	0	0	0	0	0	0
	mad2Δ	0	0	0	0	0	0	0	0	0	0	0	0

Source data Figure 8a: Quantification of the number of haploids, diploids and cells in which a mixed profile can be observed in wild type cells *mad2*Δ or *swe1*Δ cells transformed with the 2µΔNEG. Two independent experiments were performed. Experiment 1 was carried out with 4 independent colonies for each background and also carried two candidates transformed with an empty vector that were measured only on day 1 and day 4. Experiment 2 was performed similarly but one of the spores of mad2Δ cells trasnformed with the empty vector were haploid was lost (contamination).

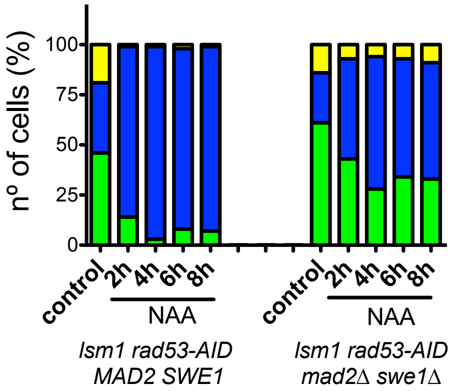

1ST EXP	*lsm1Δ rad53-AID*						*lsm1Δ rad53-AID swe1Δ mad2Δ*				
**time in NAA (h)**	**0**	**2**	**4**	**6**	**8**	**time in NAA (h)**	**0**	**2**	**4**	**6**	**8**
**G1**	46	14	3	8	7	**G1**	61	43	28	34	33
**1 nucleus**	35	85	96	90	92	**1 nucleus**	25	50	66	59	58
**2 nuclei**	19	1	1	2	1	**2 nuclei**	14	7	6	7	9
**n**	100	100	100	100	100	**n**	100	100	100	100	100
**2ND EXP**	*lsm1Δ rad53-AID*						*lsm1Δ rad53-AID swe1Δ mad2Δ*				
**time in NAA (h)**	**0**	**2**	**4**	**6**	**8**	**time in NAA (h)**	**0**	**2**	**4**	**6**	**8**
**G1**	50	17	8	4	11	**G1**	53	45	24	28	27
**1 nucleus**	41	80	90	95	84	**1 nucleus**	39	52	70	65	66
**2 nuclei**	9	3	2	1	5	**2 nuclei**	8	3	6	7	7
**n**	100	100	100	100	100	**n**	100	100	100	100	100

**AVERAGE**	*lsm1Δ rad53-AID*					*lsm1Δ rad53-AID swe1Δ mad2Δ*				
**time in NAA (h)**	**0**	**2**	**4**	**6**	**8**	**0**	**2**	**4**	**6**	**8**
**G1**	48	15,5	5,5	6	9	57	44	26	31	30
**1 nucleus**	38	82,5	93	92,5	88	32	51	68	62	62
**2 nuclei**	14	2	1,5	1,5	3	11	5	6	7	8

**SD**	*lsm1Δ rad53-AID*					*lsm1Δ rad53-AID swe1Δ mad2Δ*				
**time in NAA (h)**	**0**	**2**	**4**	**6**	**8**	**0**	**2**	**4**	**6**	**8**
**G1**	2,8	2,1	3,5	2,8	2,8	5,7	1,4	2,8	4,2	4,2
**1 nucleus**	4,2	3,5	4,2	3,5	5,7	9,9	1,4	2,8	4,2	5,7
**2 nuclei**	7,1	1,4	0,7	0,7	2,8	4,2	2,8	0,0	0,0	1,4

	ANOVA* test *(lsm1Δ rad53-AID vs lsm1Δ rad53-AID swe1Δ mad2Δ)				
**time in NAA (h)**	**0**	**2**	**4**	**6**	**8**
**G1**	ns	p<0.001	p<0.01	p<0.001	p<0.01
**1 nucleus**	ns	p<0.001	p<0.001	p<0.001	p<0.001
**2 nuclei**	ns	ns	ns	ns	ns

Source data Figure 8b. Raw data obtained in the two cell cycle kinetics performed to determine changes in the cell cycle distribution of *rad53-AID lsm1*Δ and *rad53-AID lsm1*Δ *mad2*Δ *swe1*Δ cells after treatment with NAA. Cells were fixed, stained with DAPI and classified according to morphology and number of nuclei using a 100x objective. The image on the top represents the numbers obtained in the second experiment, not included in the main text. p-values were obtained perfoming an ANOVA test, using Prism (5.0).

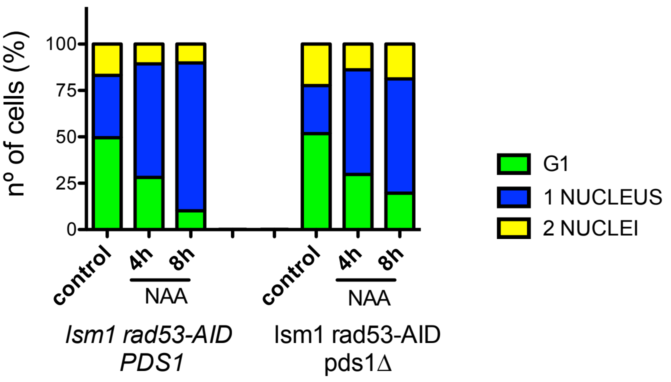

	lsm1Δ rad53-AID			*lsm1Δ rad53-AID pds1Δ*			*lsm1Δ rad53-AID*			*lsm1Δ rad53-AID pds1Δ*		
	RAW DATA			RAW DATA			%			%		
**1st experiment**	**AS**	**4H NAA**	**8H NAA**	**AS**	**4H NAA**	**8H NAA**	**AS**	**4H NAA**	**8H NAA**	**AS**	**4H NAA**	**8H NAA**
**G1**	53	29	12	58	28	22	50	28	10	52	30	20
**bud, 1 nucleus**	36	63	94	29	53	69	34	61	80	26	56	62
**bud, two nuclei**	18	11	12	25	13	21	17	11	10	22	14	19
**n**	107	103	118	112	94	112	100	100	100	100	100	100
	RAW DATA			RAW DATA			%			%		
**2nd experiment**	**AS**	**4H NAA**	**8H NAA**	**AS**	**4H NAA**	**8H NAA**	**AS**	**4H NAA**	**8H NAA**	**AS**	**4H NAA**	**8H NAA**
**G1**	76	49	20	41	45	35	44	32	18	44	41	30
**bud, 1 nucleus**	57	82	78	29	50	61	33	54	72	31	45	53
**bud, two nuclei**	41	20	11	23	15	20	24	13	10	25	14	17
**n**	174	151	109	93	110	116						

	**t-test (paired, two tails)**		
	**AS**	**4H NAA**	**8H NAA**
**G1**	0,386	0,379	0,070
**bud, 1 nucleus**	0,372	0,185	0,016
**bud, two nuclei**	0,367	0,421	0,058

	**t-test (paired, one tail)**		
	**AS**	**4H NAA**	**8H NAA**
**G1**	0,193	0,189	0,035
**bud, 1 nucleus**	0,186	0,092	0,008
**bud, two nuclei**	0,183	0,211	0,029

Source data Figure 8-Figure Supplement 8b. Raw data obtained in the two cell cycle kinetics performed to determine changes in the cell cycle distribution of *rad53-AID lsm1Δ* and *rad53-AID lsm1Δ pds1Δ* cells after treatment with NAA. Cells were fixed, stained with DAPI and classified according to morphology and number of nuclei using a 100x objective. The image on the top represents the numbers obtained in the second experiment, not included in the main text. P-values are indicated in the panel below..
