# Supplementary material for: High levels of histones promote whole-genome-duplications and trigger a Swe1WEE1-dependent phosphorylation of Cdc28CDK1
Source: eLife. 2018 Mar 27;7:e35337. doi: 10.7554/eLife.35337 (PMC5871333; doi:10.7554/eLife.35337)
Supplement: Supplementary file 1. [file elife-35337-supp1.docx]

**Supplementary File 1a. List of strains used in this study.**

| **STRAIN** | **GENOTYPE** | **SOURCE** |
| --- | --- | --- |
| DVY1 | Mat a *leu2∆0 ura3∆0 his3∆0* | This study |
| DVY1b | DVY1 2n* | This study |
| DVY2 | Mat alpha *leu2∆0 ura3∆0 his3∆0* | This study |
| DVY2b | DVY2 2n* | This study |
| DVY1-2 | Mat a/alpha leu2∆0/leu2∆0 *ura3∆0/ura3∆0 his3∆0/his3∆0* | This study |
| DVY3 | DVY1 *mad2∆:KanMX4* | This study |
| DVY4 | DVY1 pds1∆:HIS3 | This study |
| DVY5 | DVY1 *rad53K227A:KanMX4* | This study |
| DVY5b | DVY1 *rad53K227A:KanMX4 2n** | This study |
| DVY6 | DVY1 *tom1∆:KanMX4* | This study |
| DVY7 | Mat alpha *rad53-AID:KanMX4 ura3-52:URA3-GAL1pOsTyr1 sml1∆::NAT leu2∆0 his3∆0* | This study |
| DVY8 | Mat alpha *rad53-AID:KanMX4 ura3-52:URA3-GAL1pOsTyr1 sml1∆::NAT leu2∆0 his3∆0 lsm1∆HPH* | This study |
| DVY9 | DVY1 Mat a *bar1∆:HIS3* | This study |
| DVY10 | DVY5 *Mat a bar1∆:HIS3* | This study |
| DVY11 | DVY1 Mat a *DDC2:mCHERRY-HIS3* | This study |
| DVY12 | DVY1 Mat a *NUP49:GFP-HIS3* | This study |
| DVY13 | DVY1 Mat a *MAD2:GFP-HIS3 MTW1:mCHERRY-KanMX4* | This study |
| *BY smc2-8* | Mat a his3 leu2 ura3 met2 LYS2 bar∆Hyg smc2-8:TRP1 | (4) |
| DVY14 | DVY2 Mat a *MTW1:mCHERRY-KanMX4 SPC42:CFP-HIS3 prs405-GFP:TUB1* | This study |
| DVY15 | DVY1 Mat a swr1∆:KanMX4 | This study |
| DVY16 | DVY1 Mat a swe1∆:KanMX4 | This study |
| DVY17 | DVY8 Mat alpha *NUP49:GFP-HIS3* | This study |
| DVY18 | DVY8 Mat a *MAD2:GFP-HIS3 MTW1:mCHERRY-KAN* | This study |
| DVY19 | DVY7 Mat a *MAD2:GFP-HIS3 MTW1:mCHERRY-KAN* | This study |
| DVY20 | DVY7 *Mat a CSE4:MYC-URA3* | This study |
| DVY21 | DVY8 *Mat a CSE4:MYC-URA3* | This study |
| DVY22 | DVY7 Mat a *SCC1:MYC18-HPH bar1∆:HIS3* | This study |
| DVY23 | DVY8 Mat a *SCC1:MYC18-HPH bar1∆:HIS3* | This study |
| DVY24 | DVY7 Mat a *swe1∆:HIS3* | This study |
| DVY25 | DVY8 Mat a *swe1∆:HIS3* | This study |
| DVY26 | DVY7 Mat a *mad2∆:KanMX4* | This study |
| DVY27 | DVY8 Mat a *smad2∆:KanMX4* | This study |
| DVY27b | DVY8 *Mat a mad2∆LEU2 swe1∆HIS3* | This study |
| DVY28 | DVY7 Mat alpha *MTW1:mCHERRY-KanMX4 MAD2:GFP-HIS3 prs405-GFP:TUB1* | This study |
| DVY29 | DVY8 Mat alpha *MTW1:mCHERRY-KanMX4 MAD2:GFP-HIS3 prs405-GFP:TUB1* | This study |
| DVY30 | DVY7 Mat a *PDS1:HA-HIS3* | This study |
| DVY31 | DVY8 Mat a *PDS1:HA-HIS3* | This study |
| DVY32 | DVY7 Mat alpha BRN*1:HA-HIS3* | This study |
| DVY33 | DVY8 Mat alpha BRN*1:HA-HIS3* | This study |
| DVY34 | DVY8 Mat alpha *SGO1:HA-HIS3 MET3pr:CDC20* | This study |
| DVY35 | DVY8 *Mat a pds1∆:HIS3* | This study |
| DVY36 | DVY8 *Mat a NUP49:GFP-HIS3* | This study |
| DVY37 | DVY8 *Mat alpha NUP49:GFP-HIS3 HTB2:mCHERRY-URA3* | This study |

**Supplementary File 1b. List of plasmids used in this study.**

| **NAME** | **DESCRIPTION** | **SOURCE** |
| --- | --- | --- |
| prs416 | CENTROMERIC URA3 VECTOR | EUROSCARF |
| pRS426 | 2µ-URA3 VECTOR | EUROSCARF |
| CEN∆NEG | prs416 *HTA1-HTB1∆NEG* | This study |
| 2µ∆NEG | prs426 *HTA1-HTB1∆NEG* | This study |
| pRS425 | 2µ-LEU2 VECTOR | EUROSCARF |
| pRS425-HTZ1 | pRS425 HTZ1 | V. Géli´s lab |
| p67 | prs426 GAL1:HTA1 GAL10:HTB1 URA3 | (Gunjan and Verreault, 2003) |
| pMG3 | CENTROMERIC VECTOR CONTAINING A GFP:TUB1 FUSION PROTEIN INTEGRATED WITH KasI AT LEU2 LOCUS | (Gupta et al., 2002) |

**Supplementary File 1c. List of oligonucleotides used in this study.**

| **NAME AND POSITION** | **DESCRIPTION** | **SEQUENCE** |
| --- | --- | --- |
| CEN4L-F 449420 | LEFT PERICENTROMERIC REGION OF Chr IV | CCTAGGTTATCTATGCTGTCTCACC |
| CEN4L-R 449569 | LEFT PERICENTROMERIC REGION OF Chr IV | GCACTAGCCAATTTAGCACTTCTT |
| CEN4R-F 449958 | RIGHT PERICENTROMERIC REGION OF Chr IV | TCCTTCCGGTTTTATCGTCA |
| CEN4R-L 450100 | RIGHT PERICENTROMERIC REGION OF Chr IV | GATTTTCTGTTTACTCGACTTCAGG |
| CEN12L-F 150540 | LEFT PERICENTROMERIC REGION OF Chr XII | TCTGCGCCTTTCCAATAATC |
| CEN12L-R 150779 | LEFT PERICENTROMERIC REGION OF Chr XII | AAACTCTTTACGCGGGTGTG |
| CEN12R-F 150997 | RIGHT PERICENTROMERIC REGION OF Chr XII | CCTCGTACTACAAGTTTTCTCCTT |
| CEN12R-R 151218 | RIGHT PERICENTROMERIC REGION OF Chr XII | GCGTCTTGCCGTTAACAATC |
| CEN4-F 449666 | CENTROMERiC REGION OF Chr IV | ACACGAGCCAGAAATAGTAAC |
| CEN4-R 449863 | CENTROMERiC REGION OF Chr IV | GCCGCTCCTAGGTAGTGC |
| CEN12-F 150997 | CENTROMERiC REGION OF Chr XII | CGAGGTTAACATAAGAAAGAAAGAG |
| CEN12-R 150774 | CENTROMERiC REGION OF Chr XII | GTACACACCCGCGTAAAG |
| INT IV-F 358330 | INTERGENIC REGION OF Chr IV | CGAGTAACAGACGGGAAG |
| INT IV-R 358459 | INTERGENIC REGION OF Chr IV | CCTACCATCTCAATTTCTTGTTTC |
| INT XII-F 170781 | INTERGENIC REGION OF Chr XII | AAAGAAGCCAGATGAAGTGCCAGG |
| INT XII-R 170913 | INTERGENIC REGION OF Chr XII | CACCAAATAAGTACTGTGTTAAAGG |
